# Differential effects of a selective dopamine D1-like receptor agonist on motor activity and *c-fos *expression in the frontal-striatal circuitry of SHR and Wistar-Kyoto rats

**DOI:** 10.1186/1744-9081-2-18

**Published:** 2006-05-26

**Authors:** Rochellys Diaz Heijtz, F Xavier Castellanos

**Affiliations:** 1 Department of Psychiatry, New York University School of Medicine, New York VA Medical Center, 423 East 23^rd ^Street, New York, NY 10010, USA; 2 New York University Child Study Center, 215 Lexington Avenue, New York, New York 10016, USA

## Abstract

**Background:**

Molecular genetic studies suggest the dopamine D1 receptor (D1R) may be implicated in attention-deficit/hyperactivity disorder (ADHD). As little is known about the potential motor role of D1R in ADHD, animal models may provide important insights into this issue.

**Methods:**

We investigated the effects of a full and selective D1R agonist, SKF-81297 (0.3, 3 and 10 mg/kg), on motor behaviour and expression of the plasticity-associated gene, *c-fos*, in habituated young adult male Spontaneously Hypertensive Rats (SHR), the most commonly used animal model of ADHD, and Wistar-Kyoto (WKY; the strain from which SHR were derived).

**Results:**

SHR rats were more behaviourally active than WKY rats after injection with vehicle. The 0.3 mg/kg dose of SKF-81297 increased motor behaviour (locomotion, sifting, rearing, and sniffing) in both SHR and WKY rats. Total grooming was also stimulated, but only in WKY rats. The same dose increased *c-fos *mRNA expression in the piriform cortex of both strains. The 3 mg/kg dose increased sifting and sniffing in both strains. Locomotion was also stimulated towards the end of the testing period. The intermediate dose decreased total rearing in both strains, and produced a significant increase in c-fos mRNA in the striatum, nucleus accumbens, olfactory tuberculum, and in the cingulate, agranular insular and piriform cortices. The 10 mg/kg dose of SKF-81297 produced a biphasic effect on locomotion, which was characterized by an initial decrease followed by later stimulation. The latter stimulatory effect was more pronounced in SHR than in WKY rats when compared to their respective vehicle-injected groups. The 10 mg/kg dose also stimulated sifting and sniffing in both strains. Both the 3 and 10 mg/kg doses had no effect on total grooming. The 10 mg/kg dose induced significantly higher levels of *c-fos *mRNA expression in the nucleus accumbens and adjacent cortical regions (but not striatum) of SHR when compared to WKY rats.

**Conclusion:**

The present results suggest a potential alteration in D1R neurotransmission within the frontal-striatal circuitry of SHR involved in motor control. These findings extend our understanding of the molecular alterations in SHR, a heuristically useful model of ADHD.

## Background

Attention-deficit/hyperactivity disorder (ADHD) is a common behavioural disorder of childhood onset that often persists into adulthood. The neurobiology of ADHD is not well understood, but there is converging evidence implicating the catecholamine rich frontal-striatal circuitry [1]. Specifically, dysregulation of dopamine (DA) has been hypothesized in ADHD based on the potent efficacy of indirect dopaminergic agonists, such as methylphenidate, supported by in vivo evidence that methylphenidate effects are related to striatal occupancy of the dopamine transporter (DAT), by increasing extracellular dopamine levels in the striatum [2]. Molecular genetic studies have also focused on hypothesized associations between various DA related genes and ADHD, with mixed evidence regarding DAT1 and somewhat stronger results with respect to DA receptors DRD4 and DRD5 [3].

In brain, DA effects are mediated through activation of two distinct receptor families, referred to as the D1 (D1A/D1 and D1B/D5) and the D2 (D2L/S, D3 and D4) classes [4]. These receptors belong to the super-family of seven transmembrane domain receptors, which exert their biological effects via G-protein-coupled intracellular signaling pathways. The DA D1 receptors (D1R), which are expressed highly in the striatum and prefrontal cortex (PFC), may be particularly relevant for ADHD. These receptors are crucial modulators of the motor and cognitive functions mediated by the frontal-striatal circuitry [5,6], functions which are impaired in patients with ADHD. At the genetic level, preliminary evidence supports an association between ADHD and polymorphisms in D1R [7,8] or in related proteins such as calcyon [9]. In addition, D1R densities are elevated in the striatum and nucleus accumbens of spontaneously hypertensive rats (SHR) [10-12], the most commonly used genetic animal model of ADHD [13] – which is consistent with the hypothesis that D1R neurotransmission may be altered in ADHD. Accordingly, we investigated potential alterations in the motor stimulatory role of D1R in SHR, and in the normotensive Wistar-Kyoto strain (WKY; from which SHR were derived). For this purpose, we studied the motor responses to the full and selective D1R agonist, SKF-81297 (0.3, 3, and 10 mg/kg s.c.), and the expression of the plasticity-associated gene, *c-fos*, in the striatum and adjacent cortical structures, in habituated young male SHR and WKY rats. SKF-81297 was selected since it appears to be a highly selective D1R ligand in vivo, based on on its lack of inhibition of midbrain DA neurons [14], and its lack of response in DA D1 receptor-deficient mutant mice following systemic administration [15].

## Methods

### Subjects

Young adult male SHR and WKY rats (Taconic, Hudson, NY) were used. The animals arrived in the laboratory 1 week before the experiments (at 7 weeks of age) and were housed in groups of four of the same strain in standard plastic cages (Type IV Makrolon^®^), under controlled conditions of light: dark cycle (12:12 h, lights on at 07:00 h). Food and water were available *ad libitum*. The experiments were approved by the local Committee on Ethics of Animal Experimentation, Veteran Affairs Medical Hospital, Manhattan, NY.

### General behavioural procedure

Testing took place between 900 and 1600 h under dimmed light conditions. Animals were used only once to avoid carry-over effects. Prior to testing, animals were brought in their home cages to a room adjacent to the testing room, where they were weighed. Care was taken to minimize stress during transportation and handling. In order to optimize detection of agonist-induced stimulatory effects, experiments were carried out in habituated rats. Briefly, rats were removed from their home cage and placed individually in clear plexi-glass observation cages (48 × 48 cm) with wood shavings as bedding material for a period of 1 hour. Immediately after the habituation period, each rat received a single injection of either agonist or vehicle and tested for 1 hour.

### Behavioural assessment

Assessments of agonist-induced motor behaviour were carried out using a rapid time-sampling behavioural checklist technique, in a manner similar to that described previously [16]. For this procedure the behaviour of each rat was analyzed for 10-sec periods at 1-min intervals over 15 consecutive minutes using an ethologically based behavioural checklist to allow the presence or absence of the following individual behaviors (occurring alone or in combination): sniffing (flaring of nostrils with movement of vibrissae); locomotion (coordinated movement of all four limbs producing a change in location); total rearing (rearing of any form); rearing seated (front paws reaching upwards with hind limbs on floor in sitting position); rearing to wall (front paws reaching upwards onto or towards a cage while standing on hind limbs); rearing free (front paws reaching upwards away from any cage wall while standing on hind limbs); sifting (short, exploratory-type movements of the front paws through bedding material on the cage floor); total grooming (grooming of any form); stillness (motionless: awake with no behavioural changes evident, or asleep). Levels of chewing (chewing movements directed onto physical material, i.e., bedding material and/or faecal pellets, without consumption) and eating (chewing with consumption) were too low for meaningful assessment. All behavioural assessments were performed during the 5-to-20-, 25-to-40-, and 45-to-60-min time intervals after drug or vehicle administration.

### Drug procedure

(+)-6-chloro-7,8-dihydroxy-1-phenyl-2,3,4,5-tetrahydro- [1H]-3-benzazepine hydrobromide (SKF-81297, Research Biochemical and International, Natrick, MA, USA) was dissolved in isotonic saline (0.9% NaCl) vehicle. The drug was freshly made for each trial. Subcutaneous (s.c.) injections were administered into the neck in doses of 0.3, 3.0 and 10 mg/kg. Drug doses were calculated as salt weight.

### Probes and labeling

The *c-fos *mRNA was prepared from a 500 base pair long PvuII fragment of the *c-fos *cDNA as described previously [17]. The plasmid containing this cDNA fragment was linearized with appropriate restriction enzymes. ^35^S-Uridine-5'-triphosphate (UTP)-labeled RNA antisense and sense probes were transcribed *in vitro *using the appropriate template and phage RNA polymerase, purified using Probe Quant G-50 microcolumns (Amersham, NJ, USA) and checked in denaturing acrylamide gel.

### Hybridization

Expression of *c-fos *mRNA in the striatum and adjacent regions was investigated using *in situ *hybridization technique. Brains were rapidly dissected and frozen on dry ice. Coronal sections (20μm) were prepared on a cryostat and stored at -80°C until used. The *in situ *hybridization was performed as follows. The frozen tissue sections were fixed in cold 4% paraformaldehyde in 0.1 M sodium phosphate buffer pH 7.4 (PBS), for 10 min. After washing with PBS for 5 min, the sections were rinsed in DEPC-H_2_0 (5 min) and deproteinated with 0.1 M HCl for 5 min. They were then rinsed twice with PBS (3 min each) and placed into 0.25% acetic anhydride in 0.1 M triethanolamine (pH 8.0) for 20 min at room temperature; washed twice in PBS (3 min each) and dehydrated in 70%, 80% and 100% (2 min each). Sections were air dried and prehybridized (50% formamide, 50 mM Tris-HCl, pH 7.6, 25 mM ethylene-diamine-tetraacetate (EDTA), pH 8.0, 20 mM NaCl, 0.25 mg/ml yeast tRNA, 2.5 × Denhardt's solution) for 4 h at 55°C. After draining the prehybridization buffer, sections were hybridized overnight (14–16 h) in a humidified chamber at 55°C. For hybridization, labeled probe was diluted to a final concentration of 0.5 × 10^6 ^cpm/200 μl containing 50% deionized formamide (pH5), 0.3 M NaCl, 20 mM Tris-HCl (pH 7.6), 5 mM EDTA (pH 8.0), 10 mM PBS, 0.2 mM dithiothreitol, 0.5 mg/ml yeast tRNA, 0.1 mg/ml poly-A-RNA, 10% dextran sulfate, 1× Denhardt's solution. After hybridization, the slides were rinsed in 1 × standard saline citrate (SSC), 0.01% SDS (15 min); 1 × SSC, 0.01 % SDS (30 min); 50% formamide/0.5 × SSC (1 h); 1 × SSC, 0.01 % SDS (15 min) at 55°C with continuous shaking. The sections were then treated with 1μg RNase A (Roche, Nutley, NJ, USA) in RNase buffer (0.5 M NaCl, 10 mM Tris-HCl, 5 mM EDTA, ph 8.0) for 1 h at 37°C. After two additional washes in 1 × SSC, 0.01% SDS for 30 min, the sections were dehydrated in ascending alcohol series and air dried. Sections were placed against β-Max film (Amersham, NJ, USA) and stored at room temperature for 2 to 3 days. Films were developed in D19 developer for 2 min and in 1:5 dilution of Amfix fixative for 5 min. Non-specific hybridization was determined by incubating sections with the respective ^35^S-UTP-labelled sense cRNA probe for the above cDNA under identical conditions to that of the antisense RNA probe.

### Quantification

Computerized quantifications of mRNA levels were performed on film autoradiograms using an Epson Perfection 1250 scanner and NIH Image J version 1.29 (U.S. National Institutes of Health). Before performing the analysis the invert function of the program was used. In this case higher values would mean more signal (0 = black/minimum optical density and 255 = white/maximum optical density). Mean optical density was measured in the striatum, nucleus accumbens, olfactory tubercle, and various cortical regions including cingulate, agranular insular, and piriform cortices at different rostral-caudal levels. All comparisons between groups were made on sections hybridized together, under identical conditions and exposed for the same time period to β-Max film (Amersham, NJ, USA).

### Statistical analysis

Behavioural data were analyzed using either repeated measures analysis of variance (ANOVA; STRAIN × DOSE as main factors) or factorial ANOVA following a square root transformation, to allow examination of interaction effects. Statistical analysis of mRNA expression was performed using ANOVA and individual comparisons were made using Bonferroni/Dunn test. For all analyses, significance was assigned at the P < 0.05 level. All data are presented as means ± S.E.M.

## Results

### Effects of SKF-81297 on motor behaviour

#### Locomotion

**Figure 1:**
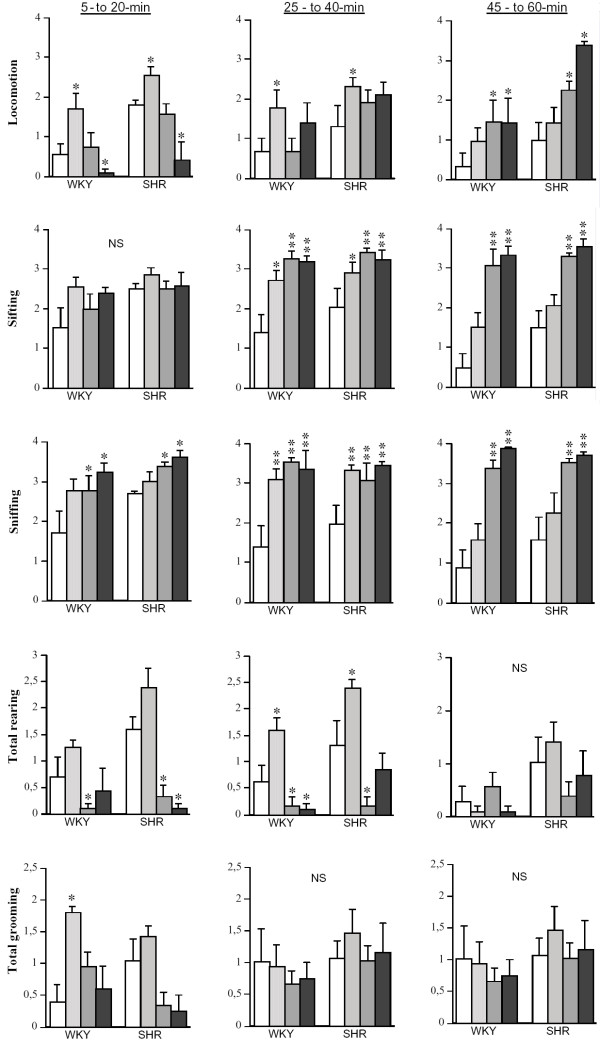
Effects of SKF-81297 on motor behaviour of SHR and WKY rats (values are shown as means ± S.E.M., n = 4–6 per each group). Doses are arrayed from vehicle to highest (0, 0.3, 3, and 10 mg/kg) for each strain from left to right. **P < 0.0001; * P < 0.05 compared to vehicle group of the same strain.

ANOVA of locomotion revealed significant main effects of STRAIN [F(1,36) = 32.46, P < 0.0001], DOSE [F(3,36) = 5.98, P < 0.01], as well as a significant TIME × DOSE [F(6,72) = 7.33, P < 0.0001] interaction. Thus, vehicle-injected SHR displayed higher levels of locomotion than vehicle-injected WKY rats throughout the testing period. In both strains, the lowest dose (0.3 mg/kg) of SKF-81297 produced a significant increase (P < 0.05) in locomotion during the 5-to-20-, and 25-to-40-min time intervals (see Fig. 1). The intermediate dose (3 mg/kg) produced an increase in locomotion only during the 45-to-60-min time interval in both strains. The higher dose (10 mg/kg) produced a biphasic effect on locomotion in both strains, which was characterized by an initial decrease during the 5-to-20-min interval followed by a significant stimulation during the last interval (45-to-60-min time interval). The later stimulatory effect was more pronounced (P < 0.05) in SHR than in WKY rats when compared to their respective vehicle-injected groups (see Fig.1).

#### Sifting

ANOVA of sifting revealed a significant main effects of STRAIN [F(1,36)= 6.67, P < 0.05], DOSE [F(3,36) = 17.16, P < 0.0001], TIME [F(2,72) = 6.60, P < 0.05], and a significant TIME × DOSE [F(6,72) = 4.49, P < 0.0001] interaction. The 0.3 mg/kg dose of SKF-81297 produced a significant increase in sifting during the 25-to-40-min interval in both SHR and WKY rats. The 3 and 10 mg/kg doses also significantly increased sifting during the middle and last intervals (i.e., 25-to-40- and 45-to-60-min) (see Fig. 1).

#### Sniffing

No significant main effects of STRAIN were found. However, there was a significant main effect of DOSE [F(3,36) = 24.31, P < 0.0001], as well as a significant TIME × DOSE [F(6,72) = 4.02, P < 0.01] interaction. SKF-81297 produced a significant dose- and time-dependent increase (P < 0.05) in sniffing in both SHR and WKY rats (see Fig. 1).

#### Total rearing

ANOVA of total rearing revealed significant main effects of STRAIN [F(1,36) = 21.61, P < 0.0001], DOSE [F(3,36) = 22.05, P < 0.0001], and TIME [F(2,72)= 3.29, P < 0.05], as well as significant DOSE × STRAIN [F(3,36) = 3.62, P < 0.05] and TIME × DOSE [F(6,72) = 4.49, P < 0.001] interactions. Thus, vehicle-injected SHR displayed significantly higher levels of total rearing than vehicle-injected WKY rats (P < 0.05) (see Fig. 1). The 0.3 mg/kg dose of SKF-81297 produced a significant increase in total rearing in both strains during the 25-to-40-min interval (see Fig. 1). The 3 and 10 mg/kg doses produced a significant decrease in total rearing during the 5-to-20-, and 25-to-40-min intervals in SHR and WKY rats (see Fig. 1). No significant effects were found during the 45-to-60-min interval. These effects of SKF-81297 on total rearing were mainly due to changes in rearing to wall (data not shown).

#### Total grooming

ANOVA of total grooming revealed significant main effects of DOSE [F(3,36) = 3.19, P < 0.05] and TIME [F(2,72) = 4.00, P < 0.05], and a significant TIME × DOSE [F(6,72) = 2.52, P < 0.05] interaction. The effects of SKF-81297 on total grooming were only observed during the 5-to-20-min interval (see Fig. 1). Thus, the 0.3 mg/kg dose produced a significant increase in total grooming in WKY rats but had no significant effect in SHR. The 3 and 10 mg/kg doses had a tendency to decrease total grooming in SHR which did not reach significance.

#### Effects of SKF-81297 on c-fos mRNA

The expression of the plasticity gene-associated gene, *c-fos*, was evaluated in the striatum and adjacent cortical regions 60 min after drug administration.

#### Striatum

**Figure 2:**
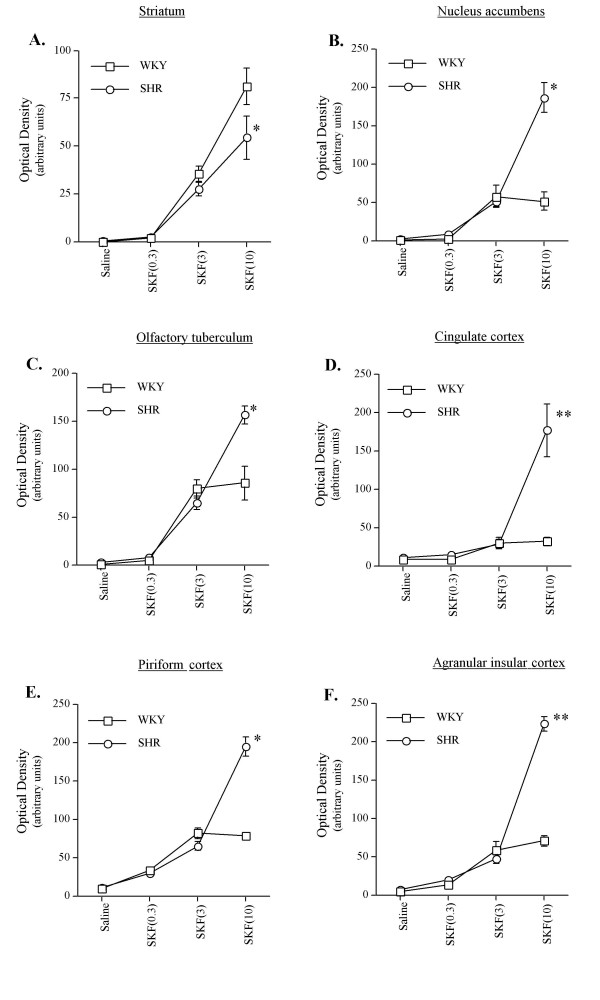
Effects of SKF-81297 (0.3, 3, and 10 mg/kg) on c-fos mRNA expression in the striatum and cortex of SHR and WKY rats (values are shown as means ± S.E.M., n = 4–6 per each group). **P < 0.0001; * P < 0.05 compared to WKY rats of the same dose. For further details see the results section.

**Figure 3:**
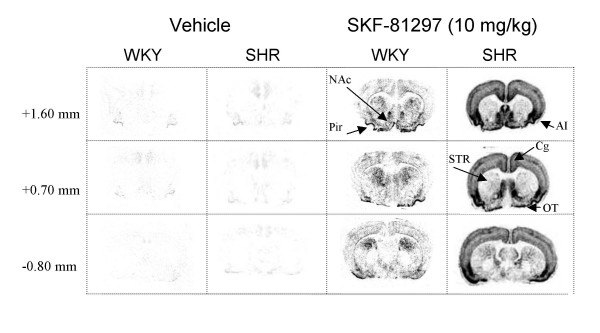
Representative autoradiograms of *c-fos *mRNA expression at different rostra-caudal levels (from top to bottom) of the striatum and adjacent cortical structures in SHR and WKY rats after vehicle or 10 mg/kg dose of SKF-81297. Coronal sections are marked "+" for anterior and "-" for posterior to bregma. STR, striatum; OT, olfactory tubercle; NAc, nucleus accumbens (shell region); AI, agranular insula cortex; Cg, cingulate cortex; Pir, piriform cortex.

Challenge with SKF-81297 had significant DOSE-dependent effects in the rostral (F(3,36) = 35.93, P < 0.0001), middle (F(3,36) = 97.37, P < 0.0001), and caudal (F(3,36) = 87.82, P < 0.0001) striatum. Further post-hoc analysis with Bonferroni/Dunn test showed that relative to vehicle, both the 3.0 and 10 mg/kg doses produced a significant (P < 0.001) increase in *c-fos *mRNA expression, especially in the dorsal striatum. ANOVA of rostral and caudal striatum revealed no significant effects of STRAIN or DOSE × STRAIN interaction. However, in the middle striatum ANOVA revealed a significant STRAIN (F(1, 36) = 7.63, P = 0.01) main effect and DOSE × STRAIN interaction (F(3,36) = 3.86, P = 0.02), with the 10 mg/kg dose inducing significantly higher levels of *c-fos *mRNA expression in WKY than in SHR rats (see Figs 2 and 3).

#### Nucleus accumbens

ANOVA revealed a significant main effect of DOSE (F(3,36) = 74.13, P < 0.0001), and STRAIN (F(1,36) = 32.76, P < 0.0001), as well as a significant DOSE × STRAIN interaction (F(3,36) = 26.34, P < 0.0001) in the shell of the nucleus accumbens. Further post-hoc analysis revealed that relative to vehicle, both the 3.0 and 10 mg/kg doses of SKF-81297 produced a significant increase (P < 0.0001) in *c-fos *mRNA, with the 10 mg/kg dose inducing significantly higher levels of *c-fos *mRNA expression in SHR rats when compared to WKY rats (see Figs. 2 and 3).

#### Olfactory tuberculum

Similar to the nucleus accumbens, ANOVA of the olfactory tuberculum revealed a significant effect of DOSE (F(3,36) = 126.05, P < 0.0001), and STRAIN (F(3,36) = 10.70, P = 0.002), as well as a significant DOSE × STRAIN interaction (F(3,36) = 9.42, P < 0.0001). Further post-hoc analysis revealed that relative to vehicle, both 3.0 and 10 mg/kg doses of SKF-81297 produced a significant increase (P < 0.0001) in *c-fos *mRNA, with the 10 mg/kg dose inducing significantly higher levels of *c-fos *mRNA expression in SHR rats when compared to WKY rats (see Figs. 2 and 3).

#### Cingulate cortex

ANOVA of the cingulate cortex revealed a significant main effect of DOSE (F(3,36)= 35.41, P < 0.0001), and STRAIN (F(1,36) = 31.25, P < 0.0001), as well as a significant DOSE × STRAIN interaction (F(3,36) = 22.20, P < 0.0001). Further post-hoc analysis revealed that relative to vehicle, both the 3.0 and 10 mg/kg doses of SKF-81297 produced a significant increase (P < 0.0001) in *c-fos *mRNA in this region, with the 10 mg/kg dose inducing significantly higher levels of *c-fos *mRNA expression in SHR rats when compared to WKY rats (see Figs. 2 and 3).

#### Agranular insular cortex

Similar to the cingulate cortex, ANOVA of the agranular insular cortex revealed a significant effect of DOSE (F(3,36) = 190.12, P < 0.0001), and STRAIN (F(3,36) = 75.22, P = 0.002), as well as a significant DOSE × STRAIN interaction (F(3,36) = 67.32, P < 0.0001). Further post-hoc analysis revealed that relative to vehicle, both 3.0 and 10 mg/kg doses of SKF-81297 produced a significant increase (P < 0.0001) in *c-fos *mRNA, with the 10 mg/kg dose inducing significantly higher levels of *c-fos *mRNA expression in SHR rats when compared to WKY rats (see Figs. 2 and 3).

#### Piriform cortex

ANOVA of this region revealed a significant main effect of DOSE (F(3,36) = 169.88, P < 0.0001), and STRAIN (F(1,36) = 30.62, P < 0.0001), as well as a significant DOSE × STRAIN interaction (F(3,36) = 29.86, P < 0.0001). Further post-hoc analysis revealed that relative to vehicle, all doses (0.3, 3, and 10 mg/kg) of SKF-81297 produced a significant increase (P < 0.0001) in *c-fos *mRNA in this region, with the 10 mg/kg dose inducing significantly higher levels of *c-fos *mRNA expression in SHR rats when compared to WKY rats (see Figs. 2 and 3). The mean ± S.E.M. values for SHR and WKY rats are as follows: 12 ± 2, 30 ± 4, 65 ± 14, 195 ± 12 and 10 ± 2, 33 ± 4, 82 ± 7, 79 ± 5, respectively, for isotonic saline vehicle, 0.3, 3, and 10 mg/kg of SKF-81297.

## Discussion

The present study was undertaken to investigate potential differences in the motor role of D1R in SHR as compared to WKY rats. We analyzed the effects of a full and selective D1-like receptor agonist, SKF-81297, on motor behaviour using an ethologically based approach. In addition, we evaluated its effects on the mRNA expression of the plasticity-associated gene, *c-fos*, in the striatum and cortex. The results suggest potential alterations in D1R neurotransmission within the frontal-striatal circuitry of SHR that is involved in motor control.

Previous studies have reported increased motor activity (i.e., horizontal and vertical activity) resulting from various D1-like receptor agonists in habituated rats, including the prototypical partial agonist, SKF-38393 [18-20]. Recent studies using full and selective agonists (SKF-81297, A-68930, and dihydrexidine) have revealed that D1R may also mediate inhibitory effects, at least with regard to motor activity under conditions of high baseline activity (e.g., non-habituated condition and d-amphetamine-induced hyperactivity) [21-24]. Importantly, the motor inhibitory effects of these full D1-like receptor agonists are not mediated by increases in stereotypy (e.g., grooming). Using an ethological approach, we confirm and extend these findings in habituated SHR and WKY rats. Similar to the effects of SKF-81297 in male Sprague-Dawley rats [22], the 10 mg/kg dose of SKF-81297 induced a biphasic effect on locomotion, which was characterized by an initial inhibition (i.e., during the 5-to-20-min interval) followed by later stimulation (i.e., during the 45-to-60-min interval) in SHR and WKY rats. The later stimulatory effects were more pronounced in SHR than in WKY rats. Consistent with our previous findings in male Sprague-Dawley rats [22], the decrease in locomotion during the initial inhibitory phase was not due to an increase in rearing or grooming because these parameters were reduced during this interval. This indicates that the initial inhibitory effect of this dose is mediated by a general decrease in locomotor drive. In contrast to previous observations in Sprague-Dawley rats, the intermediate dose (3 mg/kg) of SKF-81297 was less effective in stimulating locomotion than the 10 mg/kg dose. However, the intermediate dose was very effective in stimulating sifting and sniffing in both strains. Similar to Sprague-Dawley rats, the 0.3 mg/kg dose of SKF-81297 increased locomotion in both strains during the initial time period of testing. Because this lower dose also increased sniffing and rearing, it would suggest that this low dose of SKF-81297 heightened exploratory activity in both SHR and WKY rats.

In rodents, the meso-striatal and meso-limbic DAergic pathways are known to modulate spontaneous and DA-induced motor activity. In particular, descending influences from the nucleus accumbens via the ventral pallidum to the mesencephalic locomotor region provide a link between limbic and motor regions [25]. Neuroanatomical studies have demonstrated that D1R are highly expressed in the striatum, nucleus accumbens and olfactory tubercle, with somewhat lower concentrations in the frontal cortex [4]. Peripheral administration of a D1-like receptor agonist is known to induce a robust expression of immediate early genes (e.g., *c-fos*) in intact habituated rats, especially in the nucleus accumbens, olfactory tubercle and cortex [26]. This induction of striatal *c-fos *mRNA occurs only in medium-sized spiny neurons and can be dose-dependently correlated with increases in motor activity. Moreover, bilateral intra-accumbal injection of SKF-38393 induces a dose-related increase in motor activity [27]. In the present study, we also found a dose-dependent induction of *c-fos *mRNA in the striatum, nucleus accumbens, olfactory tubercle, and various cortical regions including cingulate, agranular insular, and piriform cortices in SHR and WKY rats. Although there were no strain differences in *c-fos *mRNA expression at the lower doses (0.3 and 3 mg/kg) of SKF-81297, the effects of the 10 mg/kg dose were highly strain dependent. Thus, in the shell of the nucleus accumbens (but not core region, data not shown), olfactory tuberculum and cortex *c-fos *mRNA expression was strikingly more pronounced in SHR than in WKY rats. It is worthwhile noting that this high dose of SKF-81297 (10 mg/kg) has failed to induce c-fos expression in the brain of D1R mutant mice (-/-) [28], whereas their wild-type control mice showed induction of c-fos and other transcription factors in the striatum neocortex, and other brain sites as described for this and other D1-like agonists in the rat. Moreover, D1R mutant mice do not respond to the locomotor stimulatory effects of SKF-81297 (see [15]). Thus, suggesting that the behavioural and cellular effects of the 10 mg/kg dose of SKF-81297 observed in the present study are mediated via activation of D1R.

The observed differences in c-fos mRNA expression between SHR and WKY rats could be due to strain differences in D1R density. For example, several authors have demonstrated increased expression of D1R binding sites in cortex, striatum, nucleus accumbens, and olfactory tuberculum of SHR [10-12], which could be a consequence of disturbances in DA uptake, storage and/or metabolism in SHR (see [29]). However, the observed strain differences in *c-fos *mRNA expression after SKF-81297 cannot be ascribed entirely to differences in D1R binding, because in the dorsal striatum *c-fos *mRNA expression (see above) was found to be more pronounced in WKY rats than in SHR. Moreover, the greatest effect of the 10 mg/kg dose of SKF-81297 on c-fos mRNA expression was observed in areas of the cortex that have lower density of D1R [4]. Therefore, cortical changes in c-fos mRNA expression could reflect downstream consequences of striatal D1R activation since the striatum receives input from (and sends reciprocal signals to) many cortical areas ([6]). There are also other possibilities that could explain the above behavioural differences and changes in *c-fos *mRNA expression after SKF-81297. One possibility is that there may be specific alterations at the level of the D1R-coupling mechanisms in different dopaminergic pathways in SHR. In the brain, D1R are known to couple to G_s_-like binding proteins, e.g., G_olf_-alpha [30], thereby stimulating adenylyl cyclase activity. Although D1R are highly expressed in the striatum, nucleus accumbens, olfactory tubercle and PFC (albeit at lower levels), there appears to be a clear segregation of their down-stream pathways. G_olf_-alpha is expressed highly in striatum, and is almost absent in PFC. The G_s_-alpha, by contrast, is expressed highly in PFC, while nucleus accumbens and olfactory tubercle have both isoforms [30]. Recent studies examining the functional coupling of D1R have found that G_olf_-alpha knockout mice do not show motor stimulatory effects to either SKF-81297 or the psychostimulant cocaine [30]. In addition, these mice do not display cocaine-induced *c-fos *mRNA expression in striatum. Alternatively, the above behavioural and molecular differences between SHR and WKY rats could be mediated by alterations in the expression of calcyon, a DA D1R interacting protein (DRD1IP), which shifts the effector coupling of D1R (i.e., from G_s _to G_q _coupling) to stimulate a calcium signaling pathway, without influencing the D1R-adenyl-cAMP pathway [31-33]. This effect requires a priming step involving activation of heterologous G_q_/_11 _protein-coupled receptors. Interestingly, polymorphisms of DRD1IP have been associated with both inattentive and hyperactive/impulsive subtypes of ADHD [9]. The expression of calcyon mRNA in the rat brain has been previously described in Sprague-Dawley rats [34]. Calcyon is highly expressed in the medial PFC and nucleus accumbens, with relatively low expression in striatum. It has been suggested that calcyon may interact with D5R, because it contains a region similar in sequence to the core calcyon binding domain of D1R. Thus, alterations in calcyon expression could affect the function of both D1R and D5R. It is worthwhile noting that D5R share high sequence homology and pharmacological profile with D1R [4] and, therefore, could contribute to the behavioural effects of SKF-81297. Further studies are required to investigate whether there are alterations in the D5R expression and/or coupling mechanisms of D1R in SHR.

Grooming behaviour has long been associated with activation of D1-class receptors in brain [16,20]. Thus, peripheral injection of DA D1-like receptor agonists elicits grooming behaviour that can be blocked by a selective D1-like receptor antagonist (SCH-23390). Moreover, congenic D1R knockouts (but not congenic D2R knockouts) show attenuation in grooming behaviour [16]. It was originally assumed that D1R play an exclusive role in regulating grooming on the basis of their high expression (relative to the D5R) in brain regions implicated in this behaviour such as the dorsolateral striatum [35]. However, new findings indicate that both D1R and D5R appear to be involved in the expression of grooming [16]. These findings are consistent with anatomical studies demonstrating modest D5R expression in discrete areas of the striatum, where they may play a role in processing signals relating to motor function. In the present study, grooming was induced in WKY rats by the lowest dose of SKF-81297 tested (0.3 mg/kg), similar to that reported previously [19,22]. However, the same dose had no significant effects in SHR. The higher doses (3 and 10 mg/kg) of SKF-81297 had no significant effects in WKY, but they had a tendency to decrease grooming in SHR during the initial testing interval. It is worthwhile mentioning that these higher doses significantly stimulated other behaviors (e.g., sifting, sniffing, rearing and locomotion) in SHR, which may compete with the expression of other behaviors such as grooming. Previous studies on the effects of SCH-23390, a selective D1-like receptor antagonist, on novelty-induced grooming behaviour have shown that SHR are less sensitive to the suppressive effects of this drug when compared to WKY rats [36]. Taken together, the results suggest a potential alteration in D1R/D5R neurotransmission in SHR possibly through primary effects within the dorsal striatum, an area involved in grooming behaviour.

To the best of our knowledge, this is the first study that has investigated the effects of a full and selective D1-like receptor agonist on motor behaviour of SHR and WKY rats. However, recent studies have investigated the effects of a cocktail of quinpirole, a DA D2-like receptor agonist, and SKF-38393, a partial D1-like receptor agonist on jaw movements in SHR and WKY rats [37,38]. These authors found that with regards to jaw movements, postsynaptic D2-like/D1-like receptors in SHR are hyposensitive when compared to those of WKY rats. Others have demonstrated that SHR are less sensitive to the motor stimulatory effects of DA D2-like receptor agonists (e.g., LY141865) as well as the mixed D1-like/D2-like receptor agonist, apomorphine [39]. Taken together with the present study, the available data indicate: (i) potential disturbances not only in D1R/D5R neurotransmission but also the D2-class of receptors in SHR, and (ii) that DA D1-like/D2-like receptors of SHR can be found in either hyper- or hyposensitive states depending upon the specific motor behavioural parameters studied. Thus, there may exist an imbalance between DA D1- and D2- classes of receptors and/or related intracellular signalling mechanisms in SHR in distinct neuronal microcircuits involved in motor control [40].

## Conclusion

In this study we analyzed the effects of a full and selective D1-like receptor agonist, SKF-81297, on motor behaviour of SHR and WKY rats. In addition, we evaluated its effects on the mRNA expression of the plasticity-associated gene, *c-fos*, in striatum and adjacent cortical areas. We found that SHR were more sensitive to the locomotor stimulatory effects of SKF-81297 when compared to WKY rats, especially at the highest dose tested (10 mg/kg). Interestingly, the same dose of SKF-81297 induced a more pronounced increase in *c-fos *mRNA expression in cortical areas and nucleus accumbens in SHR than in WKY rats, but less in the dorsal striatum. In contrast, SHR were less sensitive to the stimulatory effects of this drug on grooming behavior. These results suggest a potential alteration in D1R/D5R neurotransmission within the frontal-striatal circuitry of SHR involved in motor control. Future work should focus on further characterizing potential strain differences in D1R/D5R receptor intracellular signaling mechanisms and/or interacting proteins (e.g., calcyon) in the mesolimbic, mesocortical, and nigrostriatal DA pathways, and how they may relate to hyperactivity, impulsivity and inattention of SHR. In addition, as previously suggested by the dynamic developmental theory of ADHD, it will be of great importance to investigate how environmental factors (e.g., stress) modulate the above DA pathways and expression of the behavioural deficits in SHR [41].

## Competing interests

The author(s) declare they have no competing interests.

## Authors' contributions

RDH designed and carried out the study, conducted statistical analyses, and drafted the manuscript. FXC participated in the overall study design and helped draft the manuscript.
